# Endoscopy-assisted muscle-sparing Latissimus Dorsi muscle flap harvesting for partial breast reconstruction

**DOI:** 10.1186/s12893-020-00853-1

**Published:** 2020-08-27

**Authors:** Jeeyeon Lee, Jin Hyang Jung, Wan Wook Kim, Chan Sub Park, Ryu Kyung Lee, Ho Yong Park

**Affiliations:** Department of Surgery, School of Medicine, Kyungpook National University, Kyungpook National University Chilgok Hospital, Daegu, Republic of Korea

**Keywords:** Breast, Reconstruction, LD muscle, Endoscopy

## Abstract

**Background:**

Using the Latissimus dorsi (LD) muscle flap is one of the popular surgical technique for breast reconstruction. However, usually, long postoperative scar was remained on donor site which does not have disease. The authors applied the endoscopy-assisted surgery to harvest the LD muscle flap for breast reconstruction.

**Methods:**

From July 2018 to July 2019, five consecutive patients with breast cancer underwent partial mastectomy with endoscopy-assisted LD muscle flap reconstruction. The clinic-pathologic factors were analyzed and the cosmetic outcomes were assessed with breast shape, scarring of breast and back. A 4–6 cm of lateral incision (donor site scar) was designed and LD muscle was harvested under endoscopic surgery without gas inflation. And the harvested LD muscle was inserted for partial breast reconstruction after the cancer surgery was done.

**Results:**

Mean operative time was 116.4 min (range, 92–134) and there was no major postoperative complication. The satisfactory degree of cosmetic outcomes were shown better in patient’s survey than that of surgeon’s.

**Conclusions:**

The endoscopy-assisted LD muscle flap harvesting would be useful technique to eliminate a large donor site incision in partial breast reconstruction.

## Background

Breast cancer is the most common malignancy in women worldwide, and its incidence is still increasing [1]. Fortunately, the survival rates of breast cancer are also increasing, and more research focuses on the quality of life and cosmetic outcomes of breast cancer survivors [2, 3]. The surgical approach to breast cancer has followed the concept of oncoplastic surgery established by Audretsch et al., with the goals of achieving both oncologic safety and excellent cosmetic outcomes, especially the preservation of breast shape [4]. However, one limitation of the current approach to improving breast shape is the large remaining donor site scar after flap surgery for breast reconstruction.

Endoscopy-assisted surgery is used to reduce scarring in procedures involving various organs [5–8]. However, endoscopic and robotic surgery have not been commonly attempted for procedures involving the breast because of concerns that the space is too limited for smooth handling of the instruments.

Ideally, to harvest a latissimus dorsi (LD) flap, the surgical space could be secured with endoscopy and excellent cosmetic results at the donor site would be achieved. The endoscopy-assisted LD flap harvesting technique would be easily applied by a surgeon who has been trained in laparoscopy. Herein, we report the preliminary results of an endoscopy-assisted LD flap harvesting technique for partial breast reconstruction.

## Methods

**Fig. 1:**
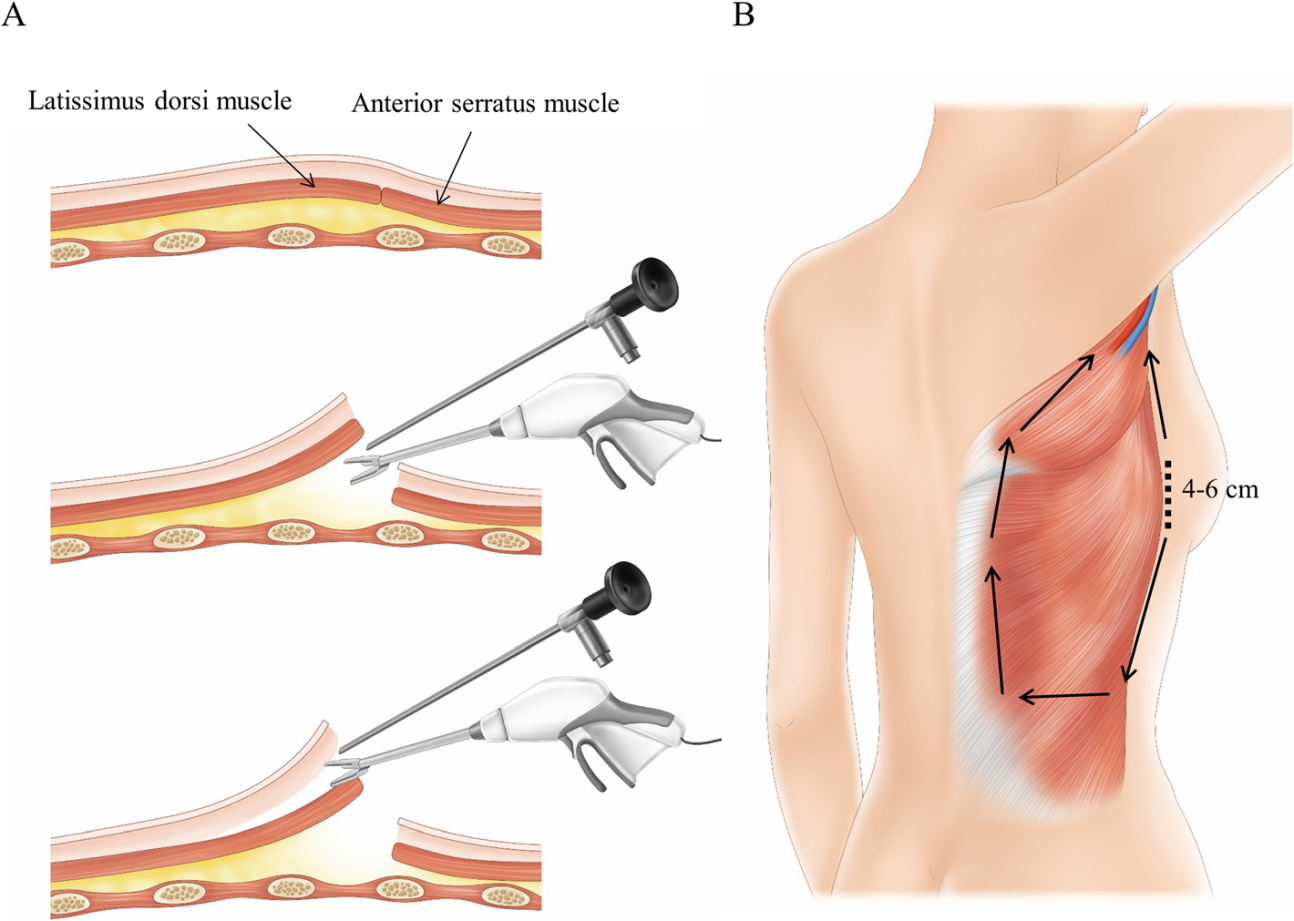
Overview of endoscopy-assisted harvesting of an LD muscle flap. **a** After the skin incision is made, the submuscular layer between the LD muscle and chest wall is dissected. Next, the subcutaneous layer between the subcutaneous fascia and LD muscle is dissected. **b** After the LD muscle is completely dissected from the chest wall and subcutaneous fat, the muscle is transected according to the required volume

From July 2018 to July 2019, five consecutive patients with breast cancer underwent partial mastectomy with endoscopy-assisted LD muscle flap reconstruction by an oncoplastic breast surgeon with 10 years of experience. The breast cancer removal procedure was conducted with conventional surgical technique except that one patient also underwent endoscopy-assisted breast surgery, and the LD muscle harvesting was performed with an endoscopic technique (Fig. 1). The inclusion criteria were: moderate-size breast (bra cup size B) and a 3 cm – 5 cm solitary mass in the upper outer portion of the breast. Patients with uncontrolled diabetes or autoimmune disease were excluded. The institutional review board of Kyungpook National University Hospital approved the study (2016–04-014), and all patients provided informed consent.

The analyzed clinical factors included age, body mass index, underlying disease, locations of breast cancer, weight of the excised breast tissue, clinical tumor size, operation time, hospital stay, and perioperative complications. The assessed pathologic factors were pathologic tumor size, axillary lymph node status, tumor stage, breast cancer characteristics, and surgical margin status. Both the surgeon and the patient assessed the cosmetic outcome using a questionnaire based on the Harvard/NSABP/RTOG Breast Cosmesis Grading Scale more than 6 months after radiotherapy. Postoperative adjuvant radiotherapy was conducted in all patients, and chemotherapy or hormone treatment was added when necessary.

### Surgical technique

The selection of each candidate for partial mastectomy with endoscopy-assisted LD muscle flap reconstruction was made after the location of the breast cancer was confirmed with preoperative mammography, ultrasonography, and breast magnetic resonance (MR) imaging.

**Fig. 2:**
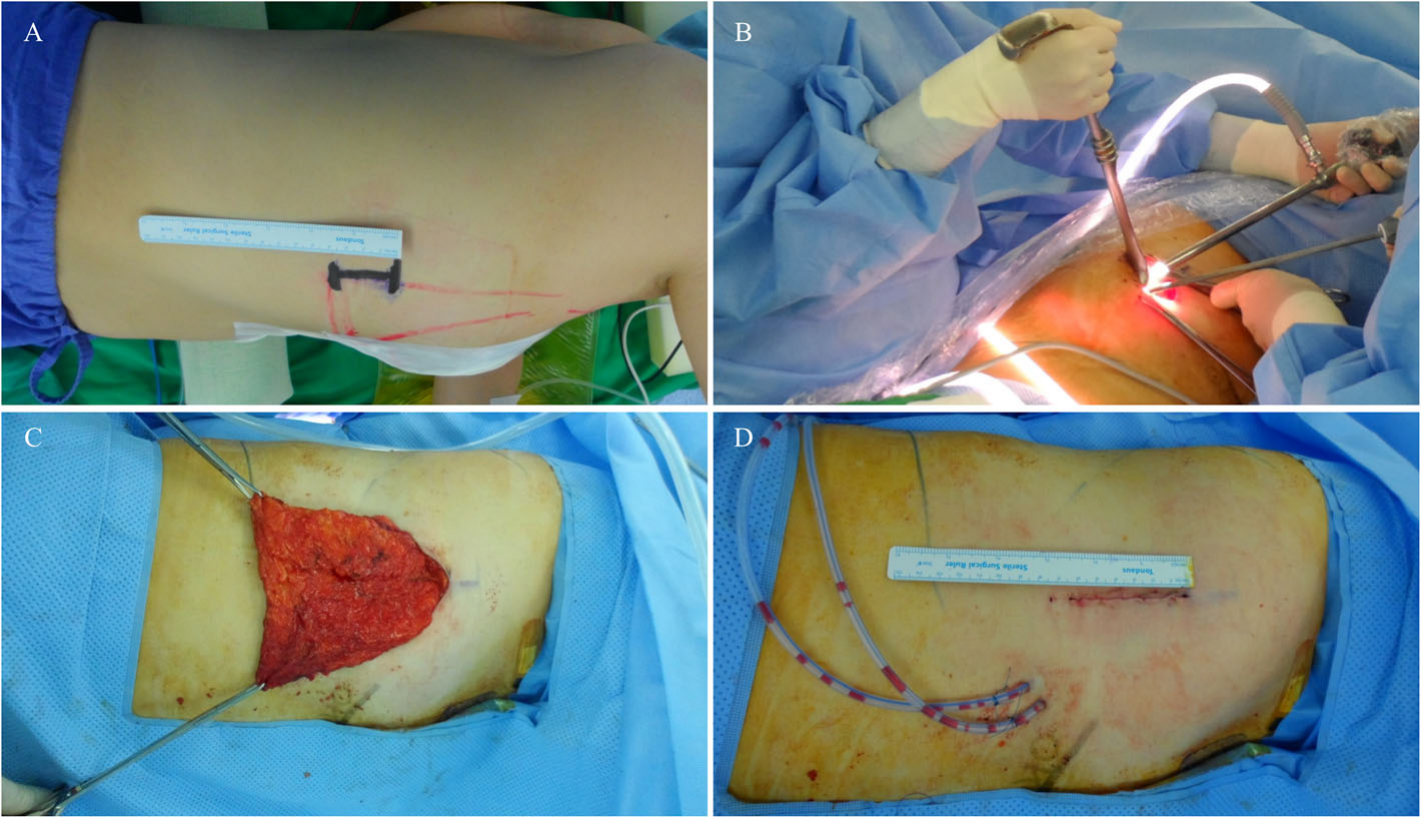
Intraoperative views of endoscopy-assisted harvesting of an LD muscle flap. **a** A 4 cm incision is made on the midaxillary line at the inframammary level. **b** The endoscopic camera, grasper, and energy device are inserted through the incision and the LD muscle is harvested. **c** Harvested LD muscle is pulled through the incision and stretched out. **d** The lateral incision (donor site scar) is closed leaving the harvested LD muscle in the cavity. However, this lateral incision was elongated 2 cm more during surgery for the better surgical field

The patient was situated on the operation table in the lateral decubitus position with the ipsilateral breast facing up. A 4–6 cm incision was made in the mid-axillary line at the level of the inframammary fold (Fig. 2a). First, the LD muscle was visualized through the incision, and the lateral portion of the LD muscle was harvested as in an open surgery procedure. Next, a small wound retractor was inserted, and the endoscopic instruments were prepared.

**Fig. 3:**
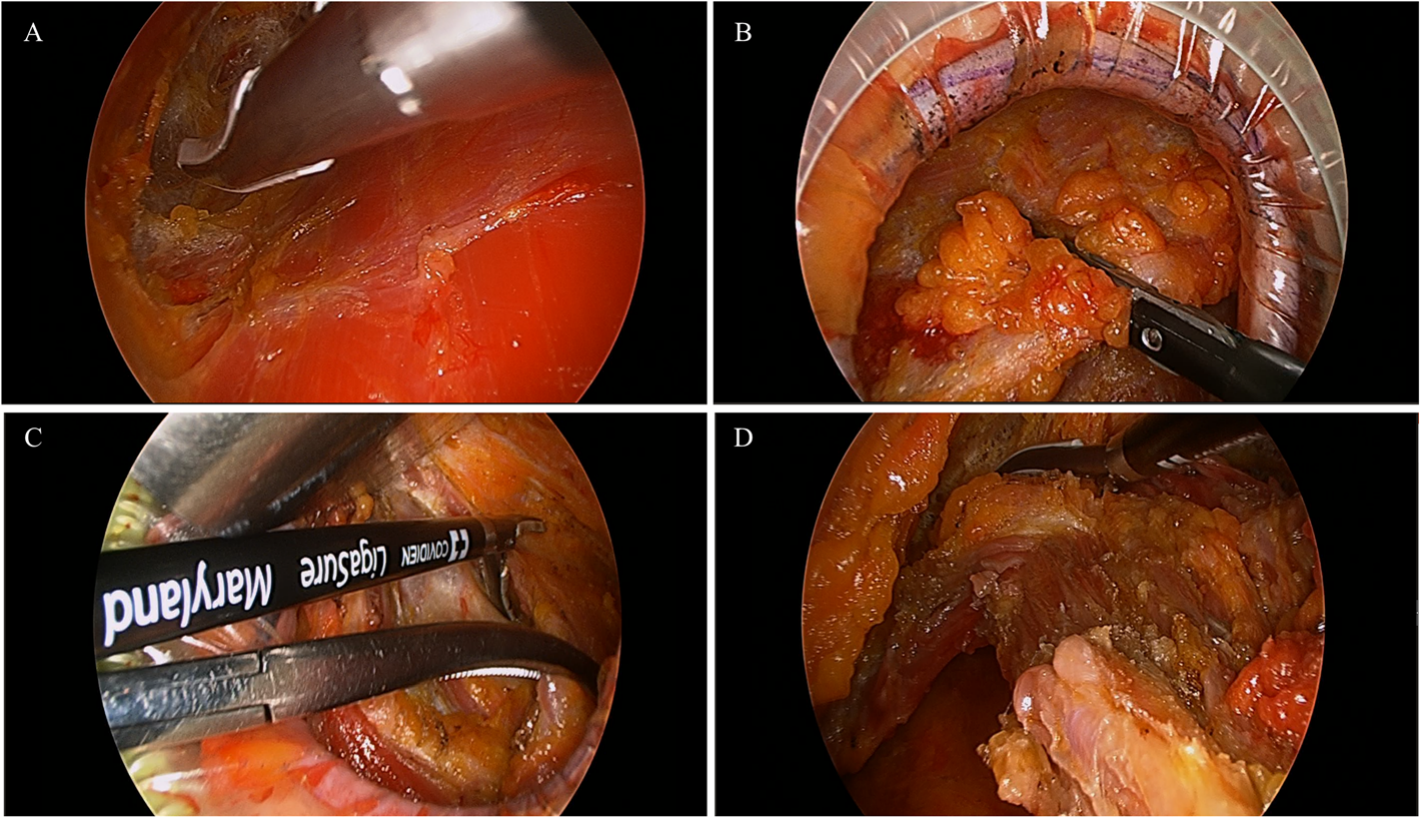
Endoscopic views of harvesting LD muscle flap. **a** After the lateral border of LD muscle is harvested with electrocautery (Bovie), the submuscular layer is dissected with energy device. **b** Then, the subcutaneous layer is also dissected with energy device and large vessels are ligated with metal clips. **c**, **d** When the subcutaneous and submuscular layers of LD muscle is sufficiently dissected, muscle boundaries are transected with energy device with traction of LD muscle

A surgical assistant elevated the overlying skin with a surgical retractor and made space to insert the endoscopic camera, grasper, and energy device (Fig. 2b). With the endoscopic camera and instruments, the subcutaneous and submuscular layers of LD muscle is sufficiently dissected with energy device (Harmonic Scalpel® or Ligasure®) (Fig. 3a, b). Because the lateral border of the LD muscle was already identified, the surgeon harvested the LD muscle according to the muscle borders and transected the inferior to upper medial border of the LD muscle when a sufficient muscle volume was harvested (Fig. 3c, d). Several surgical clips were applied to the large vessels from the chest wall to the LD muscle, or vessel sealing was done with energy device. After sufficient LD muscle was harvested to cover the breast defect (Fig. 2c), a drainage tube was inserted into the cavity. The incision was closed with 3–0 Monosyn® (B. Braun Surgical SA, Carretera de Terrassa, Rubi, Spain) for interrupted suture and 5–0 for continuous suture, leaving the harvested LD muscle in the cavity (Fig. 2d). If necessary, the volume of harvested LD muscle is calculated with water displacement method.

The patient was then turned to the supine position with both arms in abduction for the breast surgery procedure. The tumor was removed with a safety margin and the surgical margins were assessed by frozen section biopsy from the cavity in more than three different directions. Sentinel lymph node biopsy or axillary lymph node dissection was performed according to the axillary lymph node status. From the axillary incision, the harvested LD flap was pulled out and trimmed with an energy device except the thoracodorsal artery to form a thinner pedicle and reduce postoperative bulging in the axillary area. The trimmed LD muscle was fixed on the breast defect and the breast was shaped to maintain the symmetry of both breasts. After the breast shape was formed, another drainage tube was inserted in the breast and the incision was closed with Monosyn® 3–0 for interrupted suture and 5–0 for continuous suture.

## Results

The mean patient age was 56.8 years (range, 55–61 years) and the mean body mass index (BMI) was 26.9 kg/m^2^ (range, 22.9–31.3 kg/m^2^). The mean clinical tumor size was 4.0 cm (range, 2.4–6.7 cm) and the clinical stages were stage 0 (*n* = 1), IIA (*n* = 3), and IIB (*n =* 1). The mean operative time for the endoscopic-assisted LD flap harvesting was 82.6 min (range, 65–95 min) and for the partial mastectomy, sentinel lymph node biopsy, and breast reconstruction, the mean operative time was 116.4 min (range, 92–134 min); the mean weight of the removed specimen was 99.4 g (range, 59–172 g). The mean hospital stay was 11.2 days (range, 9–14 days). Additional treatment including chemotherapy, radiotherapy, and hormone treatment was determined with multidisciplinary team discussion based on the tumor stage and characteristics (Table 1). The pathologic diagnoses were invasive ductal carcinoma (*n* = 4) and ductal carcinoma in situ (*n* = 1). In all cases, the initial surgical margins were confirmed as negative with intraoperative frozen biopsy (Table 2).

**Table 1:** Clinical and operative factors of patients with breast cancer who underwent the endoscopy-assisted Latissimus dorsi muscle flap harvesting for immediate breast reconstruction

Case no.	Age (years)	BMI (kg/m^2^)	Clinical tumor size (cm)	Clinical stage	Operative time (minutes)		Weight of specimen (g)	Hospital stay (days)	Additional treatment
					Endoscopic-assisted harvesting of LD flap	Partial mastectomy, sentinel lymph node biopsy, and reconstruction			
1	50’	31.0	2.9	T2N0M0 Stage IIA	85	104	69	14	Adjuvant chemotherapy Adjuvant radiotherapy Hormone treatment
2	60’	26.1	6.7	TisN0M0 Stage 0	78	122	172	12	Adjuvant radiotherapy Hormone treatment
3	50’	23.0	3	T2N0M0 Stage IIA	90	92	59	10	Adjuvant chemotherapy Adjuvant radiotherapy
4	50’	31.3	5	T2N1M0 Stage IIB	65	134	105	11	Neoadjuvant chemotherapy Adjuvant radiotherapy
5^a^	50’	19.3	2.4	T2N0M0 Stage IIA	95	130	92	11	Adjuvant radiotherapy Hormone treatment

^a^The patient underwent both endoscopy-assisted harvesting of LD flap and endoscopic breast reconstruction

**Table 2:** Pathologic characteristics of the resected tumor in patients who underwent the endoscopy-assisted Latissimus dorsi muscle flap harvesting for immediate breast reconstruction

Case no.	Type of breast cancer	Pathologic tumor size (cm)	No. of metastatic/total removed lymph nodes	Estrogen receptor	Progesterone receptor	HER2/neu gene	Ki67 index (%)	Margin status
1	Invasive ductal carcinoma	3.5	2/7	Positive	Positive	Negative	1.5	Clear
2	Ductal carcinoma in situ	3	0/3	Positive	Positive	Negative	29.4	Clear
3	Invasive ductal carcinoma	2.3	1/8	Negative	Negative	Negative	32.1	Clear
4	Invasive lobular carcinoma	2.5	1/6	Positive	Positive	Negative	4.0	Clear
5	Invasive ductal carcinoma	1.9	0/8	Positive	Positive	Negative	14.9	Clear

Although there was no major complication such as LD flap necrosis, severe wound dehiscence, a minor complication which was donor site seroma occurred in two cases. And this postoperative seroma on donor site was managed with 2–3 times of needle aspiration under ultrasound at outpatient clinic after discharge.

**Fig. 4:**
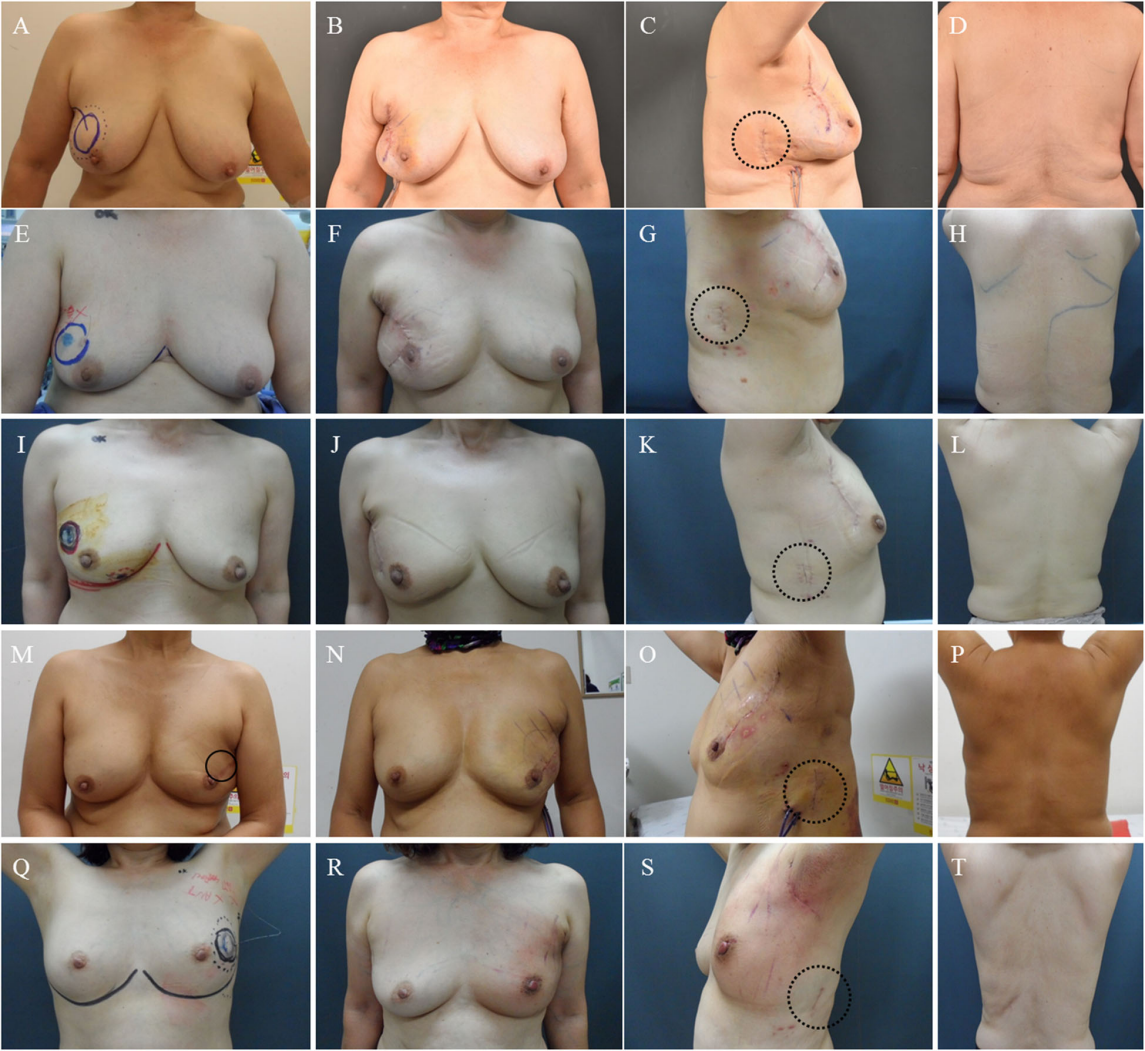
Pre- and post-operative views of four patients who received endoscopy-assisted harvesting of the LD muscle for breast reconstruction with conventional breast surgery. **a**, **e**, **i**, **m** Pre-operative views with location of the breast cancer (circles). **b**, **f**, **j**, **n** Postoperative views after 15 days. Although the breast scars are visible, the reconstructive breast shapes are well maintained. **c**, **g**, **k**, **o** Only 4–6 cm lateral incisions scars (dot circles) remain on the mid-axillary line. **d**, **h**, **l**, **p**, **t** Posterior views of all patients who received endoscopy-assisted harvesting of the LD muscle for breast reconstruction. **q-s** Pre-, post-operative, lateral views of one patient who received endoscopy-assisted harvesting of the LD muscle for breast reconstruction with endoscopy-assisted breast surgery

The cosmetic outcomes were assessed based on the Harvard/NSABP/RTOG Breast Cosmesis Grading Scale, as shown in Table 3 and Fig. 4. Because there was no remaining scar at the donor site, surgeon and patients assessed scarring at the donor site as excellent. The breast shape was assessed by the surgeon as excellent (*n* = 1), good (*n* = 2), and fair (*n =* 2) and, by the patients, as excellent (*n =* 2) and good (*n =* 3). The surgeon assessed scarring of the breast as excellent (*n =* 1), good (*n =* 2), and fair (*n =* 2), and the patients assessed scarring of the breast as excellent (*n =* 1), good (*n =* 3), and fair (*n =* 1) (Table 3).

**Table 3:** Satisfaction with cosmetic outcomes in patients with breast cancer who underwent the endoscopy-assisted Latissimus dorsi muscle flap harvesting for immediate breast reconstruction

Case no.	Follow-up period (months)	Shape of breast		Scarring of breast		Scarring of donor site	
		Surgeon	Patient	Surgeon	Patient	Surgeon	Patient
1	15.2	Good	Good	Good	Good	Excellent	Excellent
2	14.1	Good	Excellent	Fair	Fair	Excellent	Excellent
3	6.4	Fair	Good	Fair	Good	Excellent	Excellent
4	6.6	Excellent	Excellent	Good	Good	Excellent	Excellent
5	3.6	Fair	Good	Excellent	Excellent	Excellent	Excellent

## Discussion

The concept of oncoplastic surgery for breast cancer has been followed for more than two decades [9–12]. During that period, numerous surgical techniques have been developed to improve the cosmetic outcomes in patients with breast cancer. Quality of life in long-term breast cancer survivors is improved by reduced scarring and better breast shape [1–3]. Using the LD muscle flap for breast reconstruction was originally introduced for major reconstruction of the chest wall or shoulder [13, 14]. Later, Bostwick, et al. and Mendelson et al. applied this useful technique to breast reconstruction, and it remained a popular reconstructive technique for decades [15, 16]. Using the LD flap for breast reconstruction has the advantages of easy formation of breast shape and no application of dangerous structures during the surgical process except at the thoracodorsal artery. However, the biggest disadvantage was the resulting 10–15 cm back scar.

Previous attempts at generalizing endoscopic or robotic breast surgery have been hindered by hurdles such as limited space and angles [17, 18]. In contrast, the results of endoscopic or robotic surgery for harvesting the LD flap have been more frequently reported because securing space is easier in the LD cavity than in the breast [19–21]. The large scar at the LD donor site has been a major stress factor for patients with breast cancer; therefore, patient satisfaction could be markedly improved by avoidance of a 15–20 cm back scar. Endoscopic surgery requires extensive training in manipulation with the instruments rather than with the hands. Because most general surgeons learn laparoscopic and endoscopic techniques during their training, those who are skilled in conventional breast reconstruction do not find it difficult to use endoscopic surgical techniques for breast reconstruction.

**Fig. 5:**
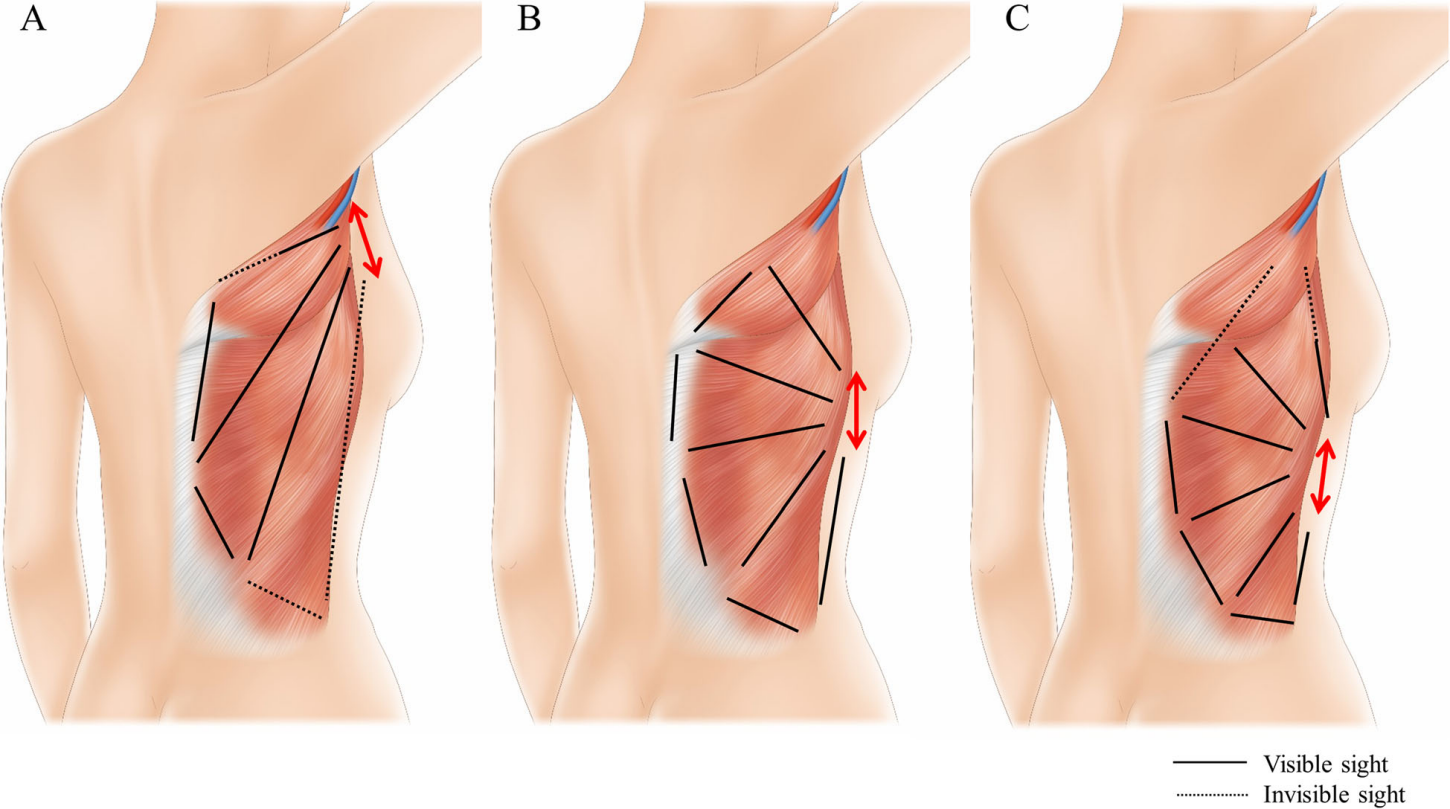
Designs of lateral incision for endoscopic harvesting of LD muscle. **a** Upper portion of lateral incision is difficult to approach to lateral and pelvic area due to narrow and straight spaces. And, due to scapula, it is also difficult to cut the upper inner border of LD muscle. **b** Middle portion of lateral incision can be a good choice to approach to every direction without major interruption. **c** Lower portion of lateral incision is difficult to identifying feeding vessels and axillary pedicle and to cut the upper inner border of LD muscle

The authors discussed a lot about the location of lateral incision (donor site scar) before performing the endoscopic harvesting of LD flap. The locations of lateral incision could be classified as upper portion which can be connected with the axillary incision, middle portion which is located on level of inframammary line, and lower portion (Fig. 4). If the lateral incision is made on upper portion, it can be connected with axillary incision and the surgical field can be secured more widely. However, if they are connected, the incision become too long even if it is hidden by arms. And if they are not connected, the it is hard to approach to lateral border of LD muscle due to too straight and narrow space, and to upper inner border of LD muscle due to scapula (Fig. 5a). When the lateral incision is designed on lower portion, it is difficult to approach to upper inner border of LD muscle and to identifying the feeding vessels which are located near to axillary area (Fig. 5c). Therefore, the authors determined that the middle portion of lateral incision to easily approach with narrow spaces to every direction. And this incision can be hidden by brassiere (Fig. 5b).

The desire to develop one’s own new surgical technique must be informed by the need to preserve the oncologic outcomes and improve on the conventional technique in every aspect. Accordingly, after we succeeded in using endoscopy for LD flap harvesting for four consecutive cases, we also tried endoscopic techniques for breast reconstruction. However, because the surgical views were not sufficiently secured, fixation of the LD flap was more difficult. Adding one small periareolar incision would make it much easier to fix the harvested flap and create a better breast shape. However, further new endoscopic skills for breast cancer should be developed to assure better cosmetic outcomes.

## Conclusion

We report our early experience with endoscopy-assisted LD muscle flap harvesting to eliminate a large donor site incision. However, in order to apply the endoscopic technique to breast surgery and reconstruction, further development and study of the approach is necessary to assure better cosmetic outcomes.

## Supplementary information

**Additional file 1: Supplemental Table1.** Cosmesis Questionnaire based on Harvard/NSABP/RTOG Breast Cosmesis Grading Scale

## Data Availability

The datasets used and/or analysed during the current study are available from the corresponding author on reasonable request.

## Abbreviations

LD: Latissimus dorsi
MR: Magnetic resonance
