# Supplementary material for: Endoscopy-assisted muscle-sparing Latissimus Dorsi muscle flap harvesting for partial breast reconstruction
Source: BMC Surg. 2020 Aug 27;20:192. doi: 10.1186/s12893-020-00853-1 (PMC7450551; doi:10.1186/s12893-020-00853-1)
Supplement: Supplementary file 1 — Additional file 1: Supplemental Table1. Cosmesis Questionnaire based on Harvard/NSABP/RTOG Breast Cosmesis Grading Scale [file 12893_2020_853_MOESM1_ESM.docx]

Supplemental table1. Cosmesis Questionnaire based on Harvard/NSABP/RTOG Breast Cosmesis Grading Scale

| Are you patient or surgeon? □ Patient □ Surgeon  * Please check your response based on the cosmetic factors. | | | |
| --- | --- | --- | --- |
|  | Shape of breast | Scarring of breast | Scarring of donor site |
| Excellent | □ | □ | □ |
| Good | □ | □ | □ |
| Fair | □ | □ | □ |
| Poor | □ | □ | □ |
